# Media representations of crimes in close relationships: Qualitative analysis of narratives in a television broadcast

**DOI:** 10.3389/fpsyg.2022.1002262

**Published:** 2022-10-14

**Authors:** Eugenio De Gregorio, Camilla Mongai, Lorenza Tiberio

**Affiliations:** ^1^Department of Life and Health Sciences, Link Campus University, Rome, Italy; ^2^Master's Program in Criminology and Psychoforensic Sciences, Department of Health Sciences, University of Genoa, Genoa, Italy; ^3^Department of Education, University of Roma Tre, Rome, Italy

**Keywords:** media communication, crime of passion, qualitative research and analysis, MAXQDA, popular criminology

## Abstract

Recent years have seen increased media attention paid to crimes committed against women by partners or former partners has grown. Crime, especially violent crime, dominates the news and the mass media. In this sense, criminology” impacts publicly the collective representation of crime. The study was motivated by the interest to understand what representations are conveyed by television broadcasts and how closely related they are to criminological theories and literature on the subject. The objective of the proposed qualitative research is to examine the media representations proposed in thematic television broadcasts, as well as the narrative profiles relating to the victims, perpetrators of the crimes and their relationships, as described in the news and by a multiplicity of roles associated with them based on kinship, friendship or professional role.

## Introduction: Crimes of passion and their media representation

Our contemporary society is characterised by violence in its various forms.

Increasingly, news stories and media stories produced by the media bring to the public’s attention more and more cases of violent behavior (persecutory acts, murders, physical, psychological and sexual violence) carried out within emotional, family and relational contexts that represent a very topical and interesting topic.

The phenomenon of violence has always existed and was considered a “normal” consequence of some relationships until a few years ago. The cultural climate has changed today, bringing the problem to light. Therefore, the community has felt the need to give a name and an explanation to this phenomenon.

The growing pervasiveness and diffusion of the mass media (including recent developments in on-demand TV and thematic channels) has helped to amplify public interest in crime news. With their realism, cinema and television dramas display contents that resemble reality and that until recently were only accessible to professionals. Consider television series with autopsies that abound or with skilled detectives who stand “with breath on their necks” of the equally skilled criminals, in what often appears as a short circuit between fiction and reality; the dramatic reciprocal influence from one level to another is evident. In other cases, video surveillance films of the most well-known and striking crimes or reconstructions featuring actors in the roles of protagonists appear to take the viewer right to the scene of the crime.

The participation (practically live) that these broadcasts allow spectators is certainly a novelty in the field of crime representation, and arouses strong emotions.

Over time, the mass media and social media have given increasing prominence to news stories regarding crimes within close relationships.

Media play an essential role in producing and reproducing of representations of reality; criminal chronicle and its protagonists are no exception. Violence against women receives prominence in conjunction with more serious facts that trigger the public agenda, events and political speeches (Easteal et al., 2015). In these processes, television is crucial.

It is common for the cases described to report stereotyped profiles of perpetrators and victims, like mainstream narratives. However, these are often one-sided narratives in which we “listen” to the voice of the journalist or editor of the television service. Rarely do we have at our disposal the reconstruction that have been made by relatives, police officers or lawyers.

According to Alvarez et al. (2021), women in Latin America most likely to carry gender stereotypes were those who tolerated harassing and abusive behavior by their partners. The evidence is interpreted in the light of socio-cultural aspects which suggest many of them have internalized traditional gender roles, believing that enduring violence within couple relationships is a necessary part of social role.

Consequently, one of the most powerful vectors of socio-cultural values can be observed in the mass media (Rollero, 2020). Communication researchers refer to cultivation theory to argue that individuals obtain information from the mass media to construct an image of reality (Gerbner et al., 2002). Furthermore, the media are identified as the primary means of conveying information and shaping individual perceptions concerning gender stereotypes and gender violence. Several studies have explored how the media constructs and reports men’s violence against women.

Rollero (2020) found that mass media cultivate images of reality and have an influence on how people describe themselves and others; by conducing semi-structured interviews with perpetrators of violence against partners, the author noted that the conceptualization of violence is not only an individual issue, but is largely influenced by the social representations conveyed through mass media.

According to Carley et al. (2008), the spectacularization of violent crime has several implications for public perceptions and social policies. The authors argue that to understand media representations of violence it is necessary to understand how they are framed. In the view of Gamson (1989) a “frame” is a central organizational idea that provide meaning to relevant events and suggests what is relevant and what is not. A frame allows you to place an event in a sort of social context.

Furthermore, the effects of this framework have repercussions both at the individual level (as deduced from the studies on individual representations) and at the social level.

## Materials and methods

The increase in media interest in crime news cases was accompanied, in particular, by a parallel interest in crimes that occur within couples, in the relationship between (former) spouses, cohabiting coupes, lovers.

The uproar aroused by these crimes lies in the fact that in almost all cases (at least those that stand out in the media crime news) the victims are women.

“Amore Criminale” is a well-known television program that has been broadcast on Rai 3 since 2007 and aims at raising public awareness, reconstructing and denouncing such situations through a filmic narration and the testimony of relatives, friends and investigators, lawyers, technical consultants, psychiatrists, criminologists. Crimes are always occurs in the context of intimate, current or terminated relationships.

The program is a *docufiction*, that is a genre of film that attempts to narrate reality through fiction. In this sense, the broadcast combines a reconstruction of the story with a documentary of its facts.

Through interviews, the lives of the murdered women are narrated, as well as the development of the couple’s relationship up to the moment of the murder.

The interviews are supported by filmed reconstructions in which there are actors who interpret some scenes from the story and documentary material (newspaper articles, television news, proceedings).

Through each episode’s narrative, the viewer is led to reflect on the social, psychological and cultural profile of the victims and murderers and the dynamics present within the couple. Thus,“folk criminology” (Popular criminology) representations of crime is created, a naive attempt (unskilled, unexperienced) to understand the crime through a process of attribution of meaning based on common sense with respect to the profiles of the perpetrator of the crime, the victim and the relationship between them (De Gregorio et al., 2017).

### Objectives and procedure

The aim of the research is to explore the explanations and interpretations, the representations of the crimes and the social actors involved in cases of murders of a woman. We will try to reconstruct the media representations as they are conveyed by a well-known thematic television program, focusing on narrative profiles of victims, perpetrators of the crimes and the relationships among themselves; these descriptions are derived from the news reported in the television broadcast and from a multiplicity of roles that are linked to them by kinship, friendship or professional role.

This exploration of media representations is based on the double level of popular and media criminology as proposed in a well-known television program entirely dedicated to crimes committed within emotional relationships.

The analysis was divided into two interconnected levels:

• The level of representations of the individual stories of the victim and the murderer;
• The level relating to the couple’s relationship and the representation of the couple in the reconstruction broadcast.

The methodological perspective that is used in this study is qualitative and interpretative based on thematic analysis (Braun and Clarke, 2006). In carrying out the thematic analysis, we resorted to “sensitizing concepts” (Blumer, 1969) which describe the conceptual foundations–metaphorically, the “bricks”–that form the basis of the analysis/interpretation process; these concepts, connected to each other in narrative themes, allow us to formulate a discourse, a narrative, in which the researcher’s interpretation must be recursively compared with the broader theoretical and conceptual frameworks. Underlining the close connection between themes and research objectives, Braun and Clarke (2006) affirm that a theme captures content considered relevant to the questions formulated in the definition of research objectives.

Based on these premises, 17 episodes of the program “Amore Criminale” were selected in the seasons broadcast between 2013 and 2018 (at the time of the research, available for free on the broadcaster’s website).

The episodes in which the victims survived the violence and attempts of oppression were excluded.

The thematic analysis was carried out for the entire episodes, including the interviews/ testimonies and the narratives of the narrator deal directly with the crime.

Besides the information contained in the transmission, no further information was acquired (from newspapers or websites), both because these sources are often included in the transmission itself and to avoid influencing the point of view artificially.

### Data management

The 17 episodes have been included in the MAXQDA program (Kuckartz and Rädiker, 2019), have been viewed and codified.

The thematic analysis of each episode was carried out by viewing each episode and fragmenting the entire movie into minimal conceptual units (narrative themes), associating each theme with codes, verbal labels that summarize its content. However, we wanted to go beyond the simple categorization of profiles and roles of the protagonists of the story, also intending to reconstruct the themes relating to their relationship as reported by the words of other narrating actors: the relatives of the victim and the perpetrator of the crime (parents, siblings, children, grandmother, grandfather, son or daughter), including the victim’s extended family (grandparents / uncles/ cousins, daughter-in-law/ son-in-law), their social networks (friends and acquaintances, neighbors, colleagues former partner of a previous relationship);

The statements coming from privileged witnesses informed about the affair and trial actors were also codified: lawyers, law enforcement agencies, public prosecutors, journalists, technicians and experts (criminologists, psychiatrists, consultants and / or the court, and forensic psychiatrists).

The coding process consists in assigning codes to the raw data that summarize the viewed content; in a subsequent phase we worked on the search for associations between narrative codes / themes to identify the representations underlying the data.

For each episode, segments have been selected that represent specific concepts/coding units; each code–where necessary–has been divided into (possible) sub-codes to further specify the content; in a subsequent phase, the codes were aggregated into sets of codes, larger conceptual units that collect the information scope of related codes pertaining to the same conceptual domain.

In addition to the information directly attributable to the profiles of the two protagonists, the elements concerning their relationship have also been codified, that is, all the discourses concerning the relational dynamics within the couple, including, for instance, the age difference between the members of the couple, the presence prior to the crime of acts of physical or psychological violence against the victim, the woman’s reactions to the violence suffered, the presence of numerous quarrels between members of the couple, the couple’s economic situation, dynamics relating to work, the presence or absence of psychiatric pathologies detected in the offender, elements connoting an emotional dependence, aspects concerning the children born from the relationship or from previous ones, the dynamics connected to the relationship that the two had with their children, the dynamics relating to the decision of the woman to move away and interrupt the relationship, the possible real or imaginary presence of a betrayal by one of the actors in the story or the presence of new relationships by one of the two, the man’s reactions to the end of the relationship.

## Results

From the analysis of the 17 stories examined in the episodes of the program “Amore Criminale,” it emerges that between these stories, 8 couples are presented who were married at the time of the murder, while 9 couples had a relationship that was not bound by marriage.

The 17 murder victims had an average of 35.5 years, in particular, they were over 30 years old in 10 cases.

The episodes examined report 3 murders in Central Italy, 7 in Southern Italy and 7 in Northern Italy.

From a first observation it emerges that the phenomenon of violence against women (more specifically the killing of one’s wife or ex-wife or partner) is a transversal phenomenon that concerns every age group, geographical area of origin of the protagonists of the story, and it is also impossible possible to draw conclusions about the status of the relationship at the time of the murder (was the relationship ended or still in progress).

When comparing the duration of relationships, one interesting fact emerges; in fact, 12 cases involved relationships that lasted 3 or more years and only 5 cases involved relationships that lasted less.

Considering the complexity of the coding of the contents relating to each story narrated, the variables that can be analyzed are numerous. In order to define the media representation of this type of crime, we limit ourselves here to examining the most salient points.

### The victims

The woman, following the violence suffered, would begin to feel particularly unhappy as reported by her close relatives and friends. In some cases, the victim of violence gradually begins to isolate himself, perhaps out of shame or because the man forces him to do so, thus hiding the real situation of the couple and the possible violence suffered from relatives and friends.

Relatives, friends, and acquaintances tell of how the woman was submissive to man, plagiarized and manipulated, confirming in a certain sense a decidedly stereotyped vision of the victim. That is, at least, the vision transmitted by the analyzed television contents. As insiders, we take into consideration that the complexity of these situations is such that it cannot be reduced to simple stereotypes (Tullio et al., 2021).

Analysing the stories in which the murder occurred after the romantic relationship between the partners ended, in 6 out of 10 stories, women felt guilty after leaving the man despite suffering the psychological and / or physical abuse, confirming the particular psychological fragility attributed to women in these situations, as has been demonstrated by academic literature and common sense.

The victim is defined as “emotionally fragile and weak” (5 stories), but also “strong and determined” (5 stories) or “energetic and vital” (10 stories). The woman is generally described as “attentive to the needs of others” (7 plots), “dreamer and idealist” (5 plots), “passionate about her work” (6 plots). The role of mother is just mentioned a few times (“an affectionate and present mother,” in 4 plots) and we imagine that this is because the role of mother and that of a woman victim of a crime would make the stereotypical representation of the profile of the victim. Finally, the characteristics of “independence” (6 plots), and a “sweet and affectionate character” (5 plots) are attributed to women.

We notice that the popular speeches about the victim concern only positive, desirable characteristics; it is well understood that they are produced by people who have been close to the woman during her lifetime and may be the result of an emotional memories.

Often, the woman is described as very attached to her family of origin (7 plots) and coming from a united and serene family environment (8 plots).

Regarding the victim, the literature also indicates vulnerability or predictability factors which represent characteristics of the woman that are related to her history, her personality organization and the situations that may have fueled the risk. However, these refer to different emotional characteristics such as aggression, anger, impulsiveness, passivity, low self-esteem, and social isolation. But once again we cannot escape the stereotypical description, typical of mass media production. In our opinion, the stereotypical description of these feelings depends on the format of the television narration which leaves no room for an in-depth analysis.

The stories told about the past of women very different from each other, more inhomogeneous than those of. Few plots (3) mention the separation of the parents, the death of one of the two or the absence of the father figure. The victim is mostly described by the figures who have been emotionally attached to her during her life, and the victim’s profile follows a typical model of the woman in this type of situation.

### The perpetrators of the crimes

As for the offender, in the 17 stories, completely different descriptions will be made with respect to his personality characteristics and life stories.

It should be borne in mind that the space dedicated to the description of the murderer’s life is less than that of the victim. This is because the narrations are mainly made by people who are emotionally related to the woman.

Psychological characteristics (character, personality, emotions, cognitive aspects) and non-objective data (socio-economic status, level of education) predominate in the representations of the protagonists, confirming that these variables most commonly suggest a stereotypic vision of the profiles described.

More specifically, the man is described as “introverted” (6 plots), “meek and calm” (7 stories), but also “arrogant and bold” (7 plots) and “liar” (4 plots). It is striking that in some cases, the man is described as a “good guy” (7 stories) and “good-looking and charming” (4 plots), as if to lessen the negative effect of the description of only undesirable traits and however less attention is paid to the physical aspect.

In this sense, media narratives offer viewers a sort of psychological identikit of the murderer, but in fact scientific literature does not refer to a set of characteristics, as according to scientific criminology it is not only a person’s character that makes him a murderer, but a set of factors found in the dynamics of the more or less remote past, in the styles of attachment with one’s caregivers as well. Only in very few cases do the narrative plots explain these factors with which interviewees and experts attempt to provide an explanation to what happened (often in a deterministic way).

Only in a few stories traumas experienced by humans are discussed as a result of the separation of parents at an early age and the subconsequent sense of abandonment.

A few cases mention alcohol and psychotropic drugs abuse, while others describe childhood and adolescence of man in terms of being bulled or witnessed violence.

A point where media narratives meet with scientific literature of a criminological nature is that the perpetrators of the crime are often described as “good guys.”

According to Karadole (2012) the perpetrators of the crime “would mostly be unsuspected in the eyes of society; the identikit of the murderer that emerges from the investigations of femicides is different from that of the stereotype of the man who is dangerous, disadvantaged, foreign and poorly integrated culturally or socially in our country” (p. 32).

These data suggest how media narratives present gaps, whether they are aware or not of the description of the perpetrator of the crime. In order to understand the man and his violent acts better, we must analyze his most profound and remote life dynamics. These dynamics are at least partially related to those present within the couple relationship.

In almost all the stories, close relatives, friends of the victim and expert technicians speak of a “sick relationship.” In narrative reconstructions, a sense of disbelief is described about the reasons in which the victim might have undertaken or continued a relationship that brought serenity and well-being.

As such, scientific literature suggests that it is rather couples in which each component brings deep needs that cannot be understood except in mutual emotional interdependence.

Our data confirm that in almost all the stories (14) the man refused to accept the separation from the woman, because he did not want to lose her. Attributed to man, only in one story is there any reference to a state of bipolar depression diagnosed prior to the homicidal act.

In 5 plots, various psychiatric pathologies are mentioned, but only in the trial phase as a possible defensive “trick” to give the accused a partial mental defect. These include Narcissistic Personality Disorder, Borderline Personality Disorder, Impulse Discontrol Disorder, and Bipolar Disorder.

The lawyers or various expert technicians who hold the role of consultants will speak about the possible presence of psychiatric pathologies.

The literature indicates that less than 10% of men who kill their partners or former companions have psychiatric problems, and therefore most violent acts in society are committed by people who are considered “healthy.”

### The relationship

The next step in our discussion is to describe the relationship between the two protagonists of the stories, the perpetrator of the crime and his victim. As mentioned before, there is a sense of disbelief described in these narrative reconstructions related to the reason in which the victim would have accepted an unhealthy relationship. There are many stories in which the women intended to distance themselves from the relationship even though they had not yet stated it explicitly. This is because they had moved away at first and then returned together with their partner. Family members and witnesses are not always aware of these movements; in fact, this information is reported in the narrations of all those who intervene in the reconstruction of the stories. There are many stories in which the woman intends to interrupt the emotional relationship. Criminological literature reports this as one of the main elements.

It was found that most perpetrators did not want to lose the woman, so they did not accept her separation from them. It is interesting to note that most of the television broadcast that deal with “emotional dependence” consult lawyers and expert technicians.

In 11 stories there are control behaviors that the man implements towards the victim during the relationship. In 5 plots the man assumes verbal behaviors aimed at denigrating, humiliating and judging the woman. These behaviors, as the literature suggests, have the purpose of nullifying the person who suffers them and whoever carries them out intends to assume a position of control and prevarication.

As another interesting fact, the isolation from the social network that men impose on women is often linked to their attitudes of control and jealousy. Here, once again, we see an enhancement of the relationship between the two protagonists.

In many stories, it is revealed how the woman progressively distances herself more and more from her family, friends and social network, becoming more and more alone and isolated.

## Conclusion

According to our findings, media representation of the history of intra-family crimes is superimposed on criminological theories. It is an overlap that leads to stereotyping the profiles of the protagonists without taking into consideration the complexities of their lives, the intricate intermeshing of their emotional relationships, and the mechanisms and reasons leading to their crime.

Additionally, to facilitate general public’s understanding of the causes of a crime, it is necessary to consider, in particular, the relationship between the victim and the perpetrator of the crime.

In this case, the murder of the partner can only be viewed as a tragic outcome of the couple’s dysfunctional relational dynamics. In a systemic perspective, victims and perpetrators of crimes are placed within a complex network of relationships and interactions. This topic cannot, however, be discussed in more detail due to the limited length of this paper.

We have analyzed stories in which representation of couple relationships and crimes are anchored to a few stereotypical elements (e.g., jealousy, possession, violence) without exploring the psychological mechanisms and processes that may underlie these manifestations explaining the complexity of relationships.

Broadening the perspective, it can be said that crime is an action by which an individual experiences himself; he confronts himself with the social, with other systems, and thus defines his own subjectivity in interaction, leaving personal traces and assuming feedback that refers to a continuous process of identity (re) elaboration (De Leo and Patrizi, 1999; De Leo et al., 2004).

The crime as the dramatic outcome of a relationship can therefore be read and interpreted through the author’s messages conveyed through the striking and disruptive action.

In examining the reactions that the man has in response to the woman’s decision to end the relationship, we see that the folk and media narratives are consistent with the scientific literature.

In scientific criminological theories, psychological, physical or economic violence is a risk factors for the murder of the partner.

The findings of this study indicate that both the media and the naive criminological narratives seem to confirm this fact, in fact, in as many as 15 plots, a form of psychological violence against women is reported.

It is possible to affirm that the media narratives and the underlying criminological theories are quite aligned with the scientific criminological theories, often because–in the years taken into consideration–a psychologist and criminologist expert in violence against women and author of several books that also deal with the themes of uxoricide and mistreatment, was present.

In several episodes, the criminologist clarified the dynamics that link murder and violence against women, in a space dedicated to her.

Several media choices were influenced by the aim of arousing interest and amazement in the viewer through highly effective narrative constructions.

These broadcasts generally present women as “victims of sick love” or “women who cannot save themselves” and often talk about a single case by generalizing some concepts to all stories of women victims of violence, thus promoting further stereotypes.

## Data availability statement

The datasets presented in this study can be found in online repositories. The names of the repository/repositories and accession number(s) can be found at: https://drive.google.com/file/d/13rqt7QuzD0LalSUq8Fxm4enSuHzDxS2v/view?usp=sharing.

## Author contributions

The article was jointly planned by ED, CM, and LT. CM took care of the data analysis and the drafting of the theoretical part. ED wrote the methodological part and supervised the data analysis. LT supervised the literature, checked the language, and wrote the conclusion. All authors contributed to the article and approved the submitted version.

## Conflict of interest

The authors declare that the research was conducted in the absence of any commercial or financial relationships that could be construed as a potential conflict of interest.

## Publisher’s note

All claims expressed in this article are solely those of the authors and do not necessarily represent those of their affiliated organizations, or those of the publisher, the editors and the reviewers. Any product that may be evaluated in this article, or claim that may be made by its manufacturer, is not guaranteed or endorsed by the publisher.
